# From Green Remediation to Polymer Hybrid Fabrication with Improved Optical Band Gaps

**DOI:** 10.3390/ijms20163910

**Published:** 2019-08-11

**Authors:** M. A. Brza, Shujahadeen B. Aziz, H. Anuar, Muataz Hazza F. Al Hazza

**Affiliations:** 1 Department of Manufacturing and Materials Engineering, Faculty of Engineering, International Islamic University of Malaysia, Kuala Lumpur, Gombak 53100, Malaysia; 2 Advanced Polymeric Materials Research Lab., Department of Physics, College of Science, University of Sulaimani, Qlyasan Street, Sulaimani 46001, Iraq; 3 Komar Research Center (KRC), Komar University of Science and Technology, Sulaimani 46001, Iraq

**Keywords:** green remediation, extract tea solution, polyphenol, copper complex, polymer hybrid, UV-vis study, XRD study, FTIR study, optical properties

## Abstract

The present work proposed a novel approach for transferring high-risk heavy metals tometal complexes via green chemistry remediation. The method of remediation of heavy metals developed in the present work is a great challenge for global environmental sciences and engineering because it is a totally environmentally friendly procedure in which black tea extract solution is used. The FTIR study indicates that black tea contains enough functional groups (OH and NH), polyphenols and conjugated double bonds. The synthesis of copper complex was confirmed by the UV-vis, XRD and FTIR spectroscopic studies. The XRD and FTIR analysis reveals the formation of complexation between Cu metal complexes and Poly (Vinyl Alcohol) (PVA) host matrix. The study of optical parameters indicates that PVA-based hybrids exhibit a small optical band gap, which is close to inorganic-based materials. It was noted that the absorption edge shifted to lower photon energy. When Cu metal complexes were added to PVA polymer, the refractive index was significantly tuned. The band gap shifts from 6.2 eV to 1.4 eV for PVA incorporated with 45 mL of Cu metal complexes. The nature of the electronic transition in hybrid materials was examined based on the Taucs model, while a close inspection of the optical dielectric loss was also performed in order to estimate the optical band gap. The obtained band gaps of the present work reveal that polymer hybrids with sufficient film-forming capability could be useful to overcome the drawbacks associated with conjugated polymers. Based on the XRD results and band gap values, the structure-property relationships were discussed in detail.

## 1. Introduction

Heavy metals entering the environment through pollution are particularly hazardous and dangerous [1]. Chemical precipitation, chemical coagulation, electrochemical technologies, ion exchange, and membrane technologies are among the various traditional methods for extraction and capturing of heavy metal elements. However, these methods are associated with numerous limitations, such as the requirement of great amounts of chemical additives that generate by-product sludge, high consumption of energy, costliness, and low efficiency when the concentrations of metals are minimal [2]. Currently, a key environmental issue is heavy metal pollution [3]. It is well known that metals possess economic importance for industrial applications and at the same time they cause the greatest environmental pollution [4]. Moreover, recent studies indicated that the group of toxic elements is both biologically and industrially important. According to up-to-date research and review papers, metalcomplexes (like Fe, Pd, Ru, Cu, Bi, Zn, etc.) were found with potential anticancer and antimicrobial activities [5,6,7,8].

Nevertheless, heavy metals do have benefits as well, with a range of nano-science and nanotechnology applications relying on heavy metal nano-particles [1]. Metal elements play a key role in chemistry as well, not only in the creation of novel advanced materials but also catalysts and reagents in chemical and related industries. According to recent research, biosorption has been considered only as an environmental remediation technique for capturing toxic and hazardous substances [2]. The cost-effectiveness and sustainability of biosorption as a strategy of bioremediation and resource management stem from the fact that it employs biomass like plant extracts and microorganisms, such as bacteria, fungi, algae and yeast [1]. Chemical precipitation has been the conventional treatment approach for the removal of heavy or trace metals, but new methods, including biosorption, neutralization, precipitation, ion exchange, and adsorption, have been recently proposed and employed on a wide basis to eliminate heavy metals from wastewater [9]. In particular, adsorption has become acknowledged as an effective and economic method for heavy metal wastewater treatment. The advantages of the adsorption process include design and operation flexibility as well as treated effluents of high quality in numerous instances [2].

The present study proposed an alternative to bio-remediation for the production of organo-metallic-based materials or metal complexes, namely, green remediation, which can capture the cations of transition metal salts (e.g., copper chloride). Earlier studies revealed that metal-complexes exhibit good absorption and cover almost the entire range of visible spectrum of light [10]. To our knowledge the combination of meal complexes with polymers may be useful for fabricating polymer composites with a controlled optical band gap. Over the last two decades, there has been noteworthy development in organic-inorganic hybrid materials, which appear to constitute a notable and definitely highly interesting research field. Even though evidentiary support now exists for the combination of organic and inorganic systems, a wide range of architectures of greater or lesser complexity of empirical implementation has been projected by chemists and physicists to address the complexity inherent in design association of materials with minimal compatibility for one another [11]. Due to their noteworthy optical, electrical and mechanical properties, polymer composites play a vital role in future material application, such as flexible electronics or photonics [12]. Various optical technologies, including solar cells and light-emitting devices, depend on the interaction between light and advanced materials. Owing to their use in sensors, optical devices and LEDs, polymers with various optical properties have recently roused a great deal of interest [13]. Adjustment of polymer optical properties can be accomplished in a simple way through controlling the dopant materials and optimising the dopant concentrations. According to the optical properties, green remediation can be considered a new approach for fabricating polymer composites with convenient optical band gaps. The findings recommend that issues like lifetime, cost and flexibility, which restrict the uses of conjugated polymers, can be addressed by using small band gap PVA with good film forming to strike a balance between cost and performance. Thus, the findings of this study can be considered as a novel step towards a new approach in polymer composites.

## 2. Results and Discussion

### 2.1. FTIR Study

**Figure 1:**
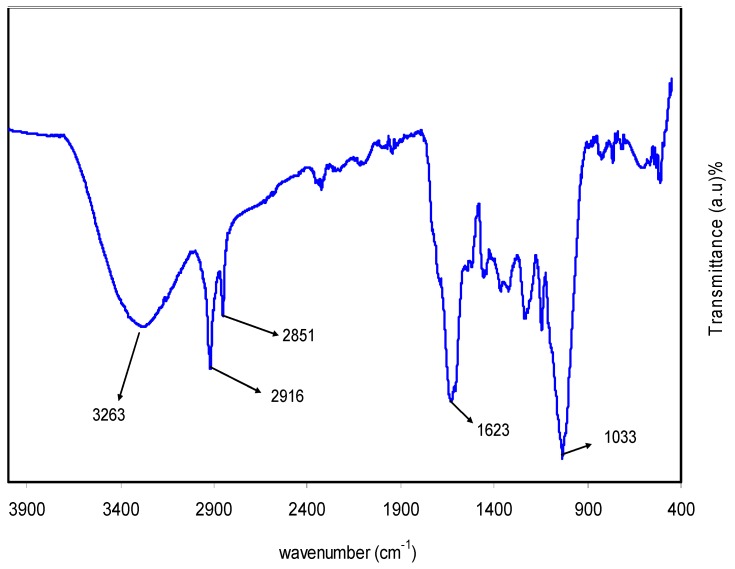
FTIR spectra of black tea leaves.

**Figure 2:**
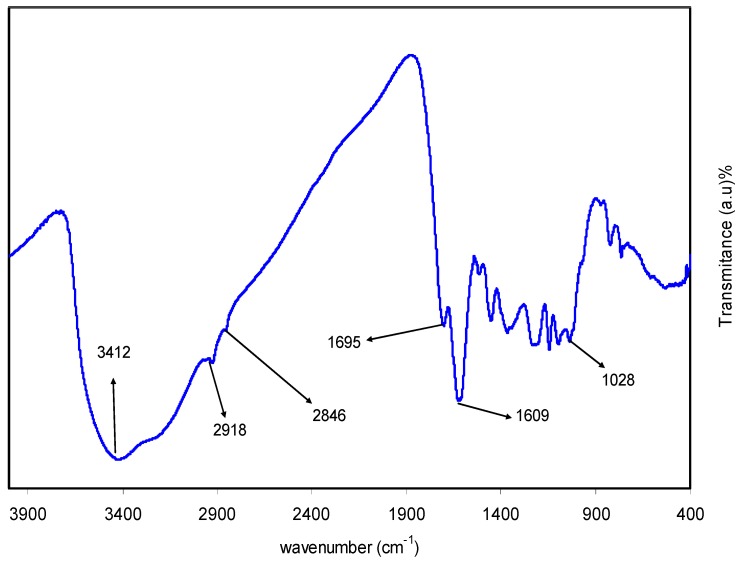
FTIR spectra of colloidal copper complex.

**Scheme 1:**
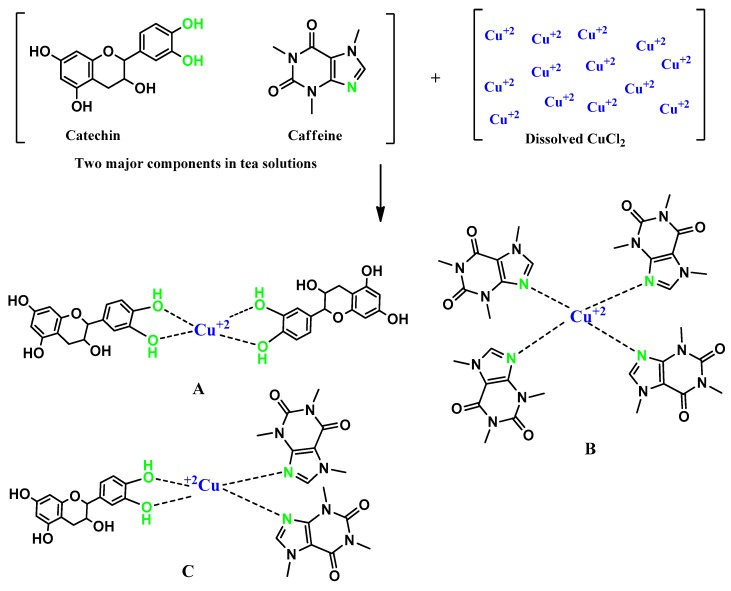
The proposed structure for the Cu metal complex formation.

The FTIR spectrum associated with black tea (BT) is illustrated in Figure 1. Several bands can be seen in the FTIR spectra. As revealed by recent research, natural dyes are receiving a great deal of attention due to their environmentally friendly as well as their deodorising, reduced toxicity, anti-allergic, anti-bacterial and anti-cancer properties [14,15]. The N-H and O-H stretching modes of polyphenols have been identified as responsible for the intense broad band occurring at 3401 cm^−1^ [16,17]. Meanwhile, a previous study claimed that O-H stretching of hydroxyl groups (alcohols, phenols and carboxylic acids) as well as N-H stretching in amines I, II and amides were associated with the broad band in the range 3490–3000 cm^−1^ [18]. The C=C stretch in aromatic ring and C=O stretch in polyphenols can be associated with a strong band at 1623 cm^−1^ [17,19]. An earlier study reported that the falls in the carbonyl frequency can be ascribed to the formation of copper complexes [20]. C-H vibration (stretch) of aliphatic groups and carboxylic acid (R-COOH) has been reported as the reason for the peaks in the range 2916–2851 cm^−1^ [17,18], while a band occurring at 1033 cm^−1^ has been attributed to the C-O stretching in amino acid [17,19]. Moreover, the band between 1750 and 1620 cm^−1^ has been assigned to the C=O vibration of bonded conjugated ketones, aldehydes, quinines and esters [18]. Additionally, as distinguished in earlier studies, the FTIR bands of tea extracts with polyphenols typically occur at 3388 cm^−1^, being referred to O-H/N-H stretching vibration, 1623 cm^−1^ (C=C stretching vibration), and 1033 cm^−1^ (C-O-C stretching vibration) [17,19,21,22]. The pattern of FTIR spectra achieved in the current work is close enough to that reported in earlier studies [18,23]. Recent studies demonstrated that changes in the spectrum of caffeine aredetected in the (1700–400) cm^−1^ regionasdepicted in Figure 1,correspondingto the stretching and binding vibrations ofthe carbonyl, imidazole, pyrimidine and methyl fragments in the caffeine [24,25]. Thus, the IR spectrum indicates that the key functional groups in tea components arecarboxylic acid, polyphenols and amino acid. There is conclusive evidence that colloidal suspensions are the outcomes of the interaction of polyphenols with proteins, caffeine and metal cations [26]. Comparing Figure 1 and Figure 2, it is obvious that the peaks in the (1700−400) cm^−1^ region almost change in Figure 2. As such, the present study is primarily geared towards demonstrating that copper colloidal attributed to copper complex can be effectively established via the FTIR technique. From the perspective of chemistry and physics, comprehending how copper complex is formed through green remediation is straightforward, owing to the rich content of polyphenols and conjugated double bonds in extract tea solution, as shown by FTIR analysis (see Figure 1), and the interaction of those components with copper salt as a method of capturing copper ions. Furthermore, the creation of Cu metal complexes among copper cations and polyphenols is confirmed by the development of colloidal suspension and green solution at the bottom and top of the beaker, respectively. The wide band appearing at 3412 cm^-1^ is a direct consequence of polyphenols in copper complex powder. As discussed above, the interaction of polyphenols with metal cations produces metal complex suspensions. The other bands observed in FTIR spectra of BT appeared again in FTIR spectra of copper complex but with shifting and decreasing intensity (see Figure 2). Goodman et al. [27], investigated the Cu complexes formation in the reaction of green and black teas with copper sulphate anhydrous (CuSO4) salt. They established the Cu complexes formation through electron paramagnetic resonance (EPR) measurements. ÓCoinceanainn et al. also studied the aluminium (III) complexes of theaflavin formation using the FTIR technique [20]. The polyphenolic compounds group unique to black tea are the theaflavins [20]. Zielinski et al. [28] also confirmed that the main components of tea are polyphenols and caffeine. The intensity of the band at 1033 cm^−1^ (see Figure 1) almost disappeared in the FTIR spectra of copper complex and shifted to 1028 cm^−1^. This is related to the fact that when coordination occurs between Cu ion and polyphenols their vibration reduces due to the attachment of copper ions and thus their weight increases. Earlier studies established that tea leaves include 10–30% (*w*/*w*) tea polyphenols and 2–4% (*w*/*w*) caffeine and all are water soluble. Moreover, they indicated that the interactions occur between caffeine, polyphenols and metal ions due to oxygen and nitrogen atoms [20,27,28,29]. According to the report of Li et al. [30], which is entitled “Black tea: chemical analysis and stability”, the main components of black tea extract solution are polyphenols and caffeine. Thus, based on previous investigation and FTIR study of the present work, the proposed complex formations of copper ions (Cu^+2^) with the polyphenols and caffeine of extract tea solution are presented in Scheme 1. From a chemistry viewpoint, various complexes can be formed among Cu metal cations and components of extract black tea solution as shown in Scheme 1. Based on previous investigations [27,28,29,30], polyphenols and caffeine are responsible for interaction with metal cations. Three expected complexations are shown in Scheme 1. It is clear from the proposed structure for the Cu-complex formation that Cu^+2^ is able to form complexation with polyphenols (Scheme 1A), caffeine (Scheme 1B), and both of them (Scheme 1C). Previous study showed the complex formation of metal cations with the polyphenols of extract black tea solution but through investigation of the EPR technique [27]. In the present work we showed the formation of metal complexes through the FTIR study. Theoptical absorption behaviour of organocopper or copper complex colloidal suspension will be addressed later on. In general, strong absorption is exhibited by organometallic-based material in visible light ranges. Differentiation of insulators, semiconductor and conducting material is dependent on light absorption, from the perspective of physics. Therefore, organocopper colloidal suspension may be better understood by conducting an optical absorption analysis.

**Figure 3:**
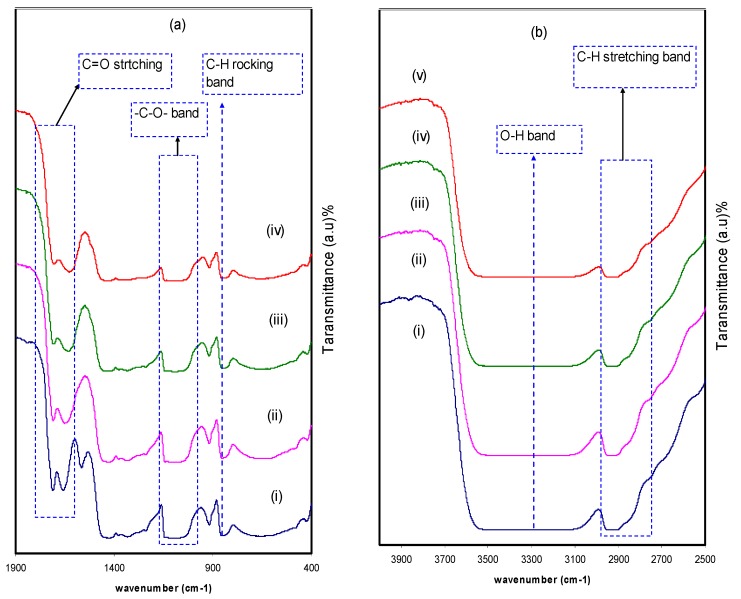
FTIR spectra of (i) PVORG0 (pure Poly (Vinyl Alcohol) (PVA) film), (ii) PVORG1, (iii) PVORG2, and (iv) PVORG3 in the region (**a**) 400 cm^−1^ to 1900 cm^−1^, and (**b**) 2500 cm^−1^ to 4000 cm^−1^.

The method of FTIR spectroscopy is essential for detection of interactions between atoms or ions in polymer electrolyte or composite systems. As a result of such interactions, the vibrational modes of the polymer electrolyte can undergo alterations [31]. Figure 3a,b illustrates the FTIR spectra of pure PVA and PVA doped with organocopper. C-H rocking of pure PVA is considered the cause of the absorption peak at 824 cm^−1^ [31]. In the case of PVA hybrid samples, this peak shifts and intensity is lowered, while addition of 45 mL of dopant material causes it to disappear almost completely. CH2 wagging has been identified as the reason for the pure PVA absorption peak at 1410 cm^−1^, while C-OH plane bending has been associated with the pure PVA absorption peak at 1316 cm^−1^ [32]. It is thus clear that the hybrid samples were associated with shifting of peaks and a significant decrease in peak intensity. Meanwhile, O-H stretching vibration of hydroxyl groups can be associated with the broad and strong absorption peak at 3340 cm^−1^ [33]. This band has a high intensity, most likely due to the strong hydrogen bonding of intra and inters type [31]. Furthermore, this band shifts and displays considerably diminished intensity in the doped samples. C=O stretching of acetate group is considered the reason for the peak at 1644 cm^−1^ in pure PVA [32], which in the case of the doped samples is shifted to smaller wave numbers. C-H asymmetric stretching vibration is associated with a band at 2908 cm^−1^ [33], which also shifts and diminishes significantly in the case of PVA hybrid samples. Moreover, the characteristic stretching vibration of –C–O– in pure PVA is reflected by the peak at 1076 cm^−1^ [34], which is shifted and loses some of its intensity in the case of the doped samples, as shown in Figure 3a. Interaction among PVA functional groups and organocopper colloidal or the adsorption of organocopper colloidal on functional groups of the host polymer is the two endorsed explanations for the band intensity fluctuating and falling. As a result, since adsorption leads to an increase in molecular weight, there is a decrease in the vibrational intensity of the functional groups [35].

### 2.2. XRD Study

**Figure 4:**
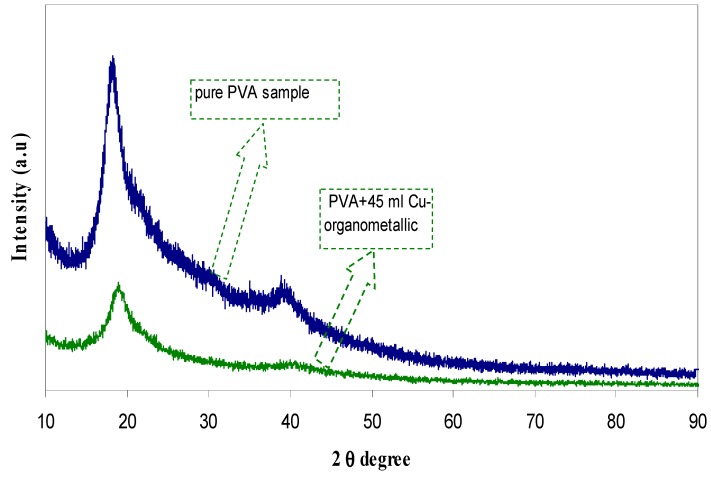
XRD pattern for pure PVA and PVA doped with 45 mL organocopper (PVORG3).

**Figure 5:**
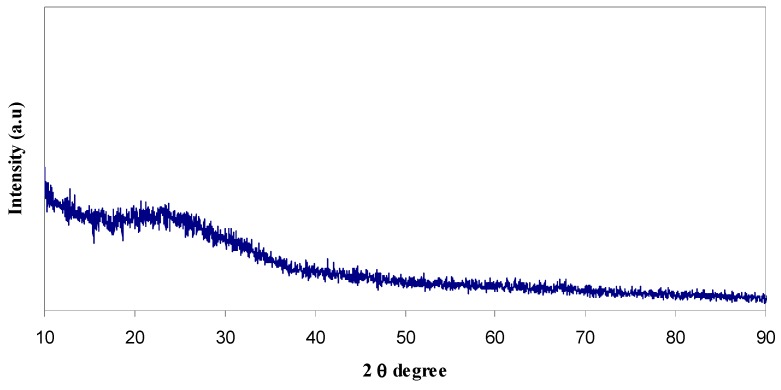
XRD pattern for synthesized copper complex as deposited.

The XRD pattern associated with pure PVA and PVA hybrid films is illustrated in Figure 4. Insight into the structure of materials and arrangement of atoms can be derived through the method of reflection of X-rays from materials. The two peaks at about 2*θ* = 20° and 2*θ* = 40° that can be discerned in the XRD pattern of pure PVA have been associated in earlier studies with the PVA crystalline domains [31,36]. It is obvious that these peaks stayed in the hybrid PVA spectra although their intensity was reduced. More specifically, the peak at 2*θ* = 40*°* nearly vanished, while the peak at 2*θ* = 20° broadened. The expansion of the amorphous phase is reflected in the broadness increase and overall reduction in the intensity of the peak at 2*θ* = 20° [35,37]. Interpretation of these findings can be based on the criterion proposed by Hodge et al. [37,38], according to which the peak intensity height is correlated with the crystallinity degree. They observed that when the dopant is added, the amorphous nature increases, which in turn cause a reduction in the XRD pattern intensity [38]. Furthermore, the XRD pattern of organocopper is shown in Figure 5, and indicates that the prepared copper complex is almost amorphous so no crystalline peaks can be seen over the entire range of 2*θ* degrees. Clearly only the hump can be observed from 2*θ* = 20° to 2*θ* = 30°. Other researchers observed such humps for as-prepared organometallic-based materials [39]. Hence, XRD analysis outcomes validate that complexation takes place between the PVA polymer and the synthesised organocopper material. Investigation of the optical band gap is necessary to analyse the fact that hybrid PVA almost amorphous. The analysis of the optical band gap that was carried out to correlate the XRD findings with material behaviour is discussed in later sections.

### 2.3. Optical Properties

#### 2.3.1. Absorption Study

**Figure 6:**
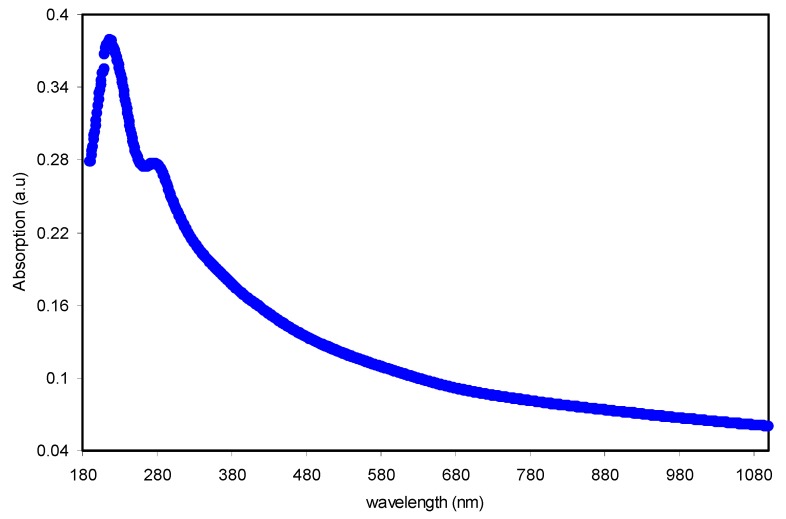
Absorptionspectra for colloidal suspension of copper complex.

The absorption spectra of a copper complex colloidal suspension are illustrated in Figure 6. It is noteworthy that the absorption covers the whole visible range, as it begins from visible range to UV range. Such absorption spectra are displayed solely by semiconductors and organometallic-based materials [40,41,42]. The formation of organocopper rather than copper nanoparticles is indicated by the fact that peaks do not occur within the range 600−800 nm [43,44,45].

Research has explored various bio-derived materials as potential heavy metal biosorbents, such as fungi, bacteria, algae, agricultural residues as well as components that can be extracted from these materials [2], especially polysaccharides (e.g., chitosan) [46,47,48,49,50,51,52]. As we reported in earlier studies, chitosan-based polymer electrolytes exhibit surface plasmonic resonance (SPR) peaks induced by copper nanoparticles at 500−800 nm [43,52].

**Figure 7:**
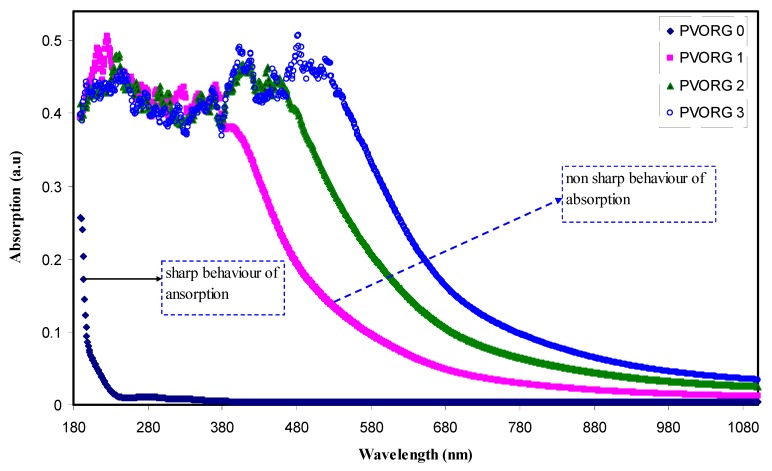
Absorption spectra of pure PVA (PVORG0) and PVA composite films.

The absorption spectra of the pure PVA polymer with their composites were studied. The results are shown in Figure 7. It can be clearly seen that the absorption spectrum of the composite samples are distributed nearly over all the significant parts of the UV-visible to near infrared regions. Furthermore, it is obvious that, owing to the absence of free electrons, pure PVA has no absorption in the visible and near infrared region. Hence, pure PVA is nearly transparent and the transfer of electrons via the band gap between the valence band and conduction band can be undertaken solely by high-energy (UV region) photons.

It is a fact that, in cases where the photon energy of the incident radiation does not exceed the energy discrepancy between two electronic states, photon absorption does not occur and the material is transparent to this radiation energy. Conversely, absorption does take place for photons with higher energy, usually in 10⁻^5^ s, and the valence electrons undergo a transition between two electronic energy levels [11]. By comparison to pure PVA, composite samples with organocopper exhibit strong absorption across the whole UV-visible region range. Furthermore, the absorption shifts to longer wavelength sides, in other words, to lower energy, as the organocopper concentration is heightened.

According to the absorption results, the organocopper-containing samples are close enough to semiconductors or conjugated double-bond polymers. There is clear evidence that, owing to the small band gap between the valence band and conduction band, absorption edges at lower energies can be exhibited solely by semiconductors or organometallic-based materials. Furthermore, it has been acknowledged that most organometallic materials possess good optical absorption and emission within a range of 600−700 nm in the wavelength [35,53]. One explanation for this may be the occurrence of orbital overlapping and help of ligands (functional groups). Therefore, energy can be transferred by electrons through the structure that turns to be responsible for the absorption spectra [35,54].

Hence, the findings of this study prove that green chemistry approaches can be employed to fabricate hybrid polymers based on polar polymers like PVA with reduced optical band gaps. It is reasonable to say that this constitutes advancement in polymer physics due to the status of polar polymers as wide band gap polymers displaying good mechanical properties and being less expensive than conjugated polymers. However, further investigation is necessary to confirm that green remediation is an alternative environmentally friendly method to other more expensive methods to fabricate organometallic-based materials and subsequently to fabricate polymer hybrids with controlled energy band gaps. It has been reported that polymer electrolytes with high ionic d.c conductivity are crucial in the application of electrochemical devices, such as supercapacitors, batteries, etc., and similarly the fabrication of polymer composites with small energy band gaps and high performance optical properties are important in the application of photonics, optoelectronic devices, and solar cell devices [55]. Thus, it is required to further enhance light-harvesting capabilities, shift absorption bands to the NIR region, widen absorption bands, and raise extinction coefficients so as to approach high-power conversion efficiency solar cells [56]. The performance of solar cells depends upon several factors, for example the absorption efficiency of the fabricated material for the solar radiation spectrum [57].

Determination of the energy band gap of crystalline and amorphous materials depends significantly on the optical absorption spectrum. The nature and value of the energy band gap can be established based on the fundamental absorption corresponding to the electron excitation from the valence band to the conduction band [58]. Furthermore, to comprehend and estimate the optical band gap of the films, it is helpful to analyse optical absorption spectra. The absorption coefficient is dependent on the reflectance and transmittance results [59]:

(1) $$\alpha =\frac{1}{t}Ln\left[\sqrt{\frac{{\left(1-R\right)}^{4}+4T^{2}R^{2}-{\left(1-R\right)}^{2}}{2TR^{2}}}\right]$$

In Equation (1), the absorption coefficient is denoted by α, transmittance is denoted by T, thickness is denoted by t, and reflectance is denoted by R. The value of T can be obtained using Beer’s law (i.e., T =10^−A^), where A is the raw absorption data. When a light ray transmits from one medium (e.g., air) into another (e.g., solid), there are various things that can occur. Some of the light ray may be reflected, some absorbed and some transmitted within the medium. The incident beam intensity to the second medium surface (I_o_) must be equal to the sum of the intensities of the reflected, absorbed and transmitted beams, designating as I_R_, I_A_ and I_T_, respectively, or

*I_O_* = *I_R_* + *I_A_* + *I_T_* (2)

**Figure 8:**
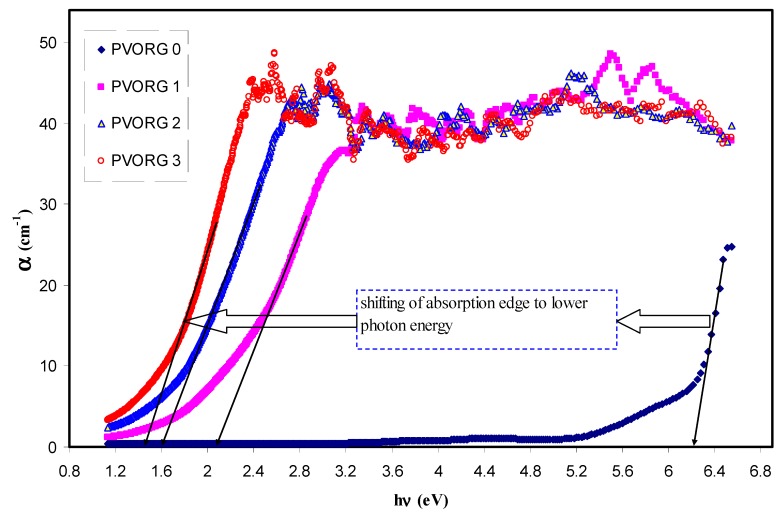
Absorption coefficient vs photon energy for pure PVA (PVORG0) and PVA composite films.

The intensity of the radiation measured in watts per square meter refers to the energy passed per unit time per unit area that is perpendicular to the propagation direction. Hence, by dividing Equation (2) by *I_o_*, one can get the following relation:

*R* + *A* + *T* = 1 (3)

where, *R*, *A*, and *T* denote, the reflectivity (*I_R_/I_o_*), absorptivity (*I_A_/I_o_*) and transmissivity *(I_T_/I_o_*), respectively, or the incident light fractions that are reflected, absorbed and transmitted, respectively. Because of that the whole incident light is either reflected, absorbed or transmitted, their total must equal one [60]. Thus, the parameter of reflectance (*R*) which is vital also for the investigation of the refractive index can be simplycalculated from Equation (3) (*R* = *1* − (*A + T*). For the aims of identifying the materials’ electronic structure, the optical absorption analysis and particularly the absorption edge has been significantly helpful. Optical absorption spectra can be indicative of the occurrence of both indirect and direct transition within the band gap [61]. Figure 8 shows the absorption coefficients versus photon energy. The values of the absorption edge are presented in Table 1. Pure PVA has an absorption edge value of around 6.26 eV, while the organocopper-containing samples reveal a wide shift to lower photon energy. Siddaiah et al. [62] reported that the value of absorption edge for pure PVA lies at 5.94. The absorption edge value of the current study for pure PVA corresponds to that attained in the study of Siddaiah et al. The wide shifting of the absorption edge can be attributed to the distribution and complexation between organocopper and PVA matrix.

There is conclusive evidence that optical band gaps are diminished by the distribution of states localised within the energy gap due to trapping the charge carriers [63]. The wide absorption edge shifting to lower photon energy (Figure 8) is close enough to those reported for inorganic and organic semiconductors. Organic semiconductors are employed extensively in electronic and optoelectronic technology, being particularly relevant for electronic and photonic devices owing to their wide range of applications [64]. Ample research has been conducted on the optical and electrical properties of metal complexes in order to derive optical materials, which may be promising candidates for optical applications [65]. The development of metallic and semiconducting materials is especially dependent on the synthesis of new charge transfer complexes. Given their uses in electronic and optical devices, investigations of the electrical and optical properties of such materials have roused significant interest [61].

A key parameter for the design of optical materials is the refractive index, which is highly informative for optical materials of higher efficiency. Refractive index modifications possess significance for controlling the optical properties of the optical material-based metal complex under investigation [65]. The design of optoelectronic devices depends greatly on accurate information about the refractive index parameter. In theory, the refractive index is a function of density and mean polarizability of medium, which alters with pressure and temperature [66]. Thus, the refractive index is among the major parameters influencing optical performance. Calculation of film refractive index can be based on reflectance and absorption, and the expression of the complex refractive index of films can take the following form:

n*_(λ)_ = n_(λ)_ + k_(λ)_ (4)

**Figure 9:**
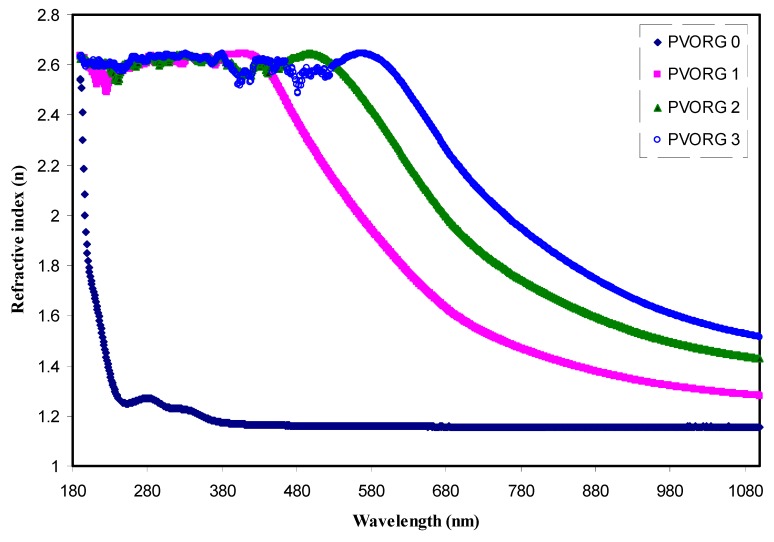
Refractive index spectra versus wavelength for pure PVA (PVORG0) and PVA composite films.

In the above, the extinction coefficient is denoted by k, while the refractive index is denoted by n. The correlation between the values of n and k is expressed as [59]:

(5) $$n=\left[\frac{\left(1+R\right)}{\left(1-R\right)}\right]+\sqrt{\frac{4\times R}{{\left(1-R\right)}^{2}}}-K^{2}$$

where *K* (*K* = *αλ*/4π*t*) is extinction coefficient in Equation (5), which is directly proportional to the absorption coefficient (*α*) and wavelength (*λ*) and inversely proportional to the thickness of the sample (*t*) [67]. The refractive index (*n*) is illustrated in Figure 9 as a wavelength function. It is obvious that doped samples are associated with higher n values, exhibiting noteworthy dispersion. As previously indicated, n value modification according to wavelength is essential for controlling the optical properties of materials, with their dispersion being highly important from the perspective of application. It is clear that n values increase with increasing organocopper, displaying noticeable dispersion within both visible and near infrared region. Due to the moderately narrow n selection, the optical applications of polymers are not as broad as those of inorganic solids. Most viable polymers have n values within the range 1.3−1.7, while inorganic materials can have n values of less than 1, as in the case of gold, or exceeding 3, as in the case of lead sulphite [68]. Studies have demonstrated that polymeric composites with extremely high n can be obtained by introducing inorganic nanoparticles, semiconductor and organometallic-based materials into a polymer matrix, which could be potentially useful for lenses, optical filters, reflectors, optical waveguides, optical adhesives, solar cells, or antireflection films [61,68].

Basically, dielectric characteristics constitute a reflection of the optical properties of a solid material [59]. The dielectric constant varies according to photon energy, suggesting that certain photon-electron interactions in the film are generated within this range of energy. Noticeable interactions on the shapes of the real and imaginary parts of the dielectric constant cause peaks to form in dielectric spectra [65]. It has been suggested that the real and imaginary parts of the dielectric constant are associated not only with refractive index values, but also with extinction coefficient values, as expressed below [69]:

(6) $$\varepsilon_{1}=n^{2}-k^{2}=\varepsilon_{\infty }-\frac{e^{2}}{4\pi C^{2}\varepsilon_{0}}\frac{N}{m^{*}}\lambda^{2}$$

**Figure 10:**
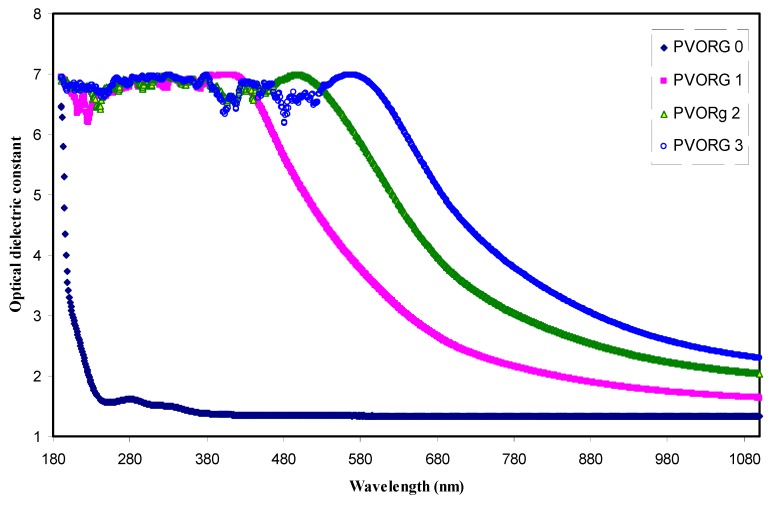
Dielectric constant spectra versus wavelength for pure PVA (PVORG0) and PVA composite films.

In Equation (6), the dielectric constant at higher wavelengths is denoted by ε_∞_, the free space dielectric constant is denoted by ε_o_, the ratio of localised electronic state density to effective mass is denoted by N/m*, the electronic charge is denoted by *e*, the optical relaxation time is denoted by τ, and the velocity of light is denoted by C. The optical dielectric constant (ε’) spectra are represented in Figure 10 in relation to wavelength for every sample, and it is clear that the increase in the copper complex concentration determines a rise in the ε’ value from 1.32 to 2.29. The increase in the density of states is the reason for this rise, as a direct correlation can exist between ε’ and the density of states within the forbidden gap of the solid polymer films [35,37].

It has been argued that the band gap has a major influence on the static dielectric constant *ε_(o)_* within long wavelengths [70]. It was in 1962 that Penn established that the optical dielectric constant and energy band gap were correlated [71], developing a model to illustrate this correlation:

*ε*_(o)_ ≈ 1 + (*ℏω_p_*/*E_o_*)^2^ (7)

**Figure 11:**
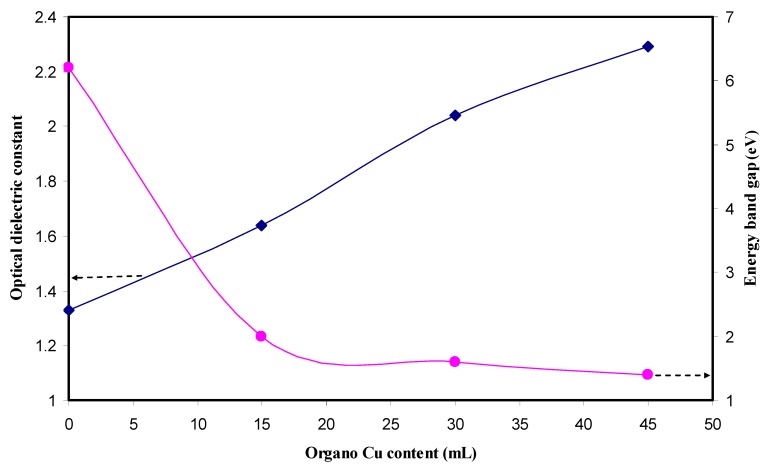
Dielectric constant and energy band gap versus organocopper content.

It is possible to link this model to the refractive index (*n*), as *ε* = *n*^2^. Hence, the expression of the Penn model can be formulated in relation to the refractive index [72]. This model makes use of dielectric constant of long wavelength because it is constant and almost plateaus [73]. The optical dielectric constant is illustrated in Figure 11 against the filler fraction, while the optical band gap against filler concentration is shown in the inset of that same figure. The density of states increases as the optical dielectric constant increases. On the other hand, the increase in the optical dielectric constant causes a reduction in the optical band gap. The design, synthesis and use of polymers with small band gap (usually *E_g_* < 2 eV) are the main aspects addressed within organic photovoltaics for the purposes of solar cell application [74]. The findings of the present study confirm that the Penn model, which maintains that the energy band gap diminishes with the increase of density of states in the band gap, is valid.

#### 2.3.2. Band Gap Study

There is limited comprehension about the electron transition in semiconducting-conducting polymers and charge transfer complexes. The incident photon confers the required energy while the phonon produces the necessary momentum [75]. According to earlier research, the optical band gap can be measured based on the optical dielectric loss, while the types of electronic transitions can be determined based on Tauc’s model, owing to the fact that the band structure of materials has little influence on the optical dielectric function. There is broad agreement that estimation of the material band structure on the whole is greatly facilitated by the use of UV-vis spectroscopy to analyse the optical dielectric function [35,43,76,77,78,79,80,81,82]. Meanwhile, the optical properties of a solid can be better comprehended through the investigation of the complex dielectric function (ε^*^ = ε_1_-i·ε_2_) that characterises the linear response of the material to an electromagnetic radiation. The optical absorption in the material is denoted by the imaginary part ε_2_, which has a close correlation with the valence (occupied) and conduction (unoccupied) bands and is calculated as [77]:

(8) $$\varepsilon_{2}(\omega )=\frac{2e^{2}\pi }{\Omega \varepsilon_{o}}\sum_{K,V,C}|\Psi_{K}^{C}|\vec{U}\cdot \vec{r}{\left|\Psi_{K}^{V}\right|}^{2}\delta (E_{K}^{C}-E_{K}^{V}-\hbar \omega )$$

In the above, the incident photon frequency and the crystal volume are respectively denoted by ω and Ω, the electron charge and free space permittivity are respectively denoted by e and ε_o_, the position vector and a vector representing the incident electromagnetic wave polarisation are respectively denoted by $\vec{r}$ and $\vec{u}$, while the conduction and valence band wave functions at k are respectively denoted by $\Psi_{k}^{v}$ and $\Psi_{k}^{c}$. The theoretical models imply that the optical dielectric constant is characterised by a complex function of frequency, the calculation of which demands large-scale computational endeavour [70,71,73,83]. From an experimental perspective, the imaginary part of the optical dielectric function (ε_2_) can be determined based on the derived refractive index and extinction coefficient and applying the relations below [43,77,78,79,80,81,82]:

(9) $$\varepsilon_{2}=2\times n\times K$$

**Figure 12:**
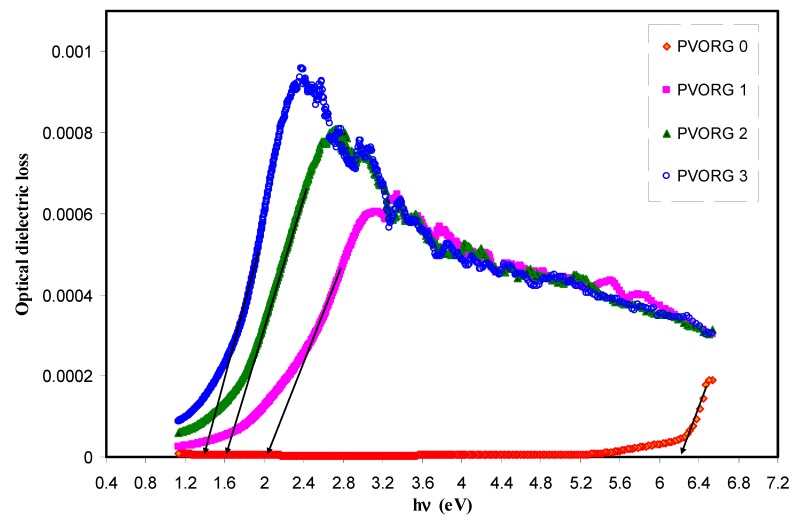
Dielectric loss spectra versus photon energy for pure PVA (PVORG0) and PVA composite films.

In Equation (9), the refractive index and the extinction coefficient are respectively denoted by n and k. Evidence has been provided by previous research that interband transitions are the direct cause of the peaks occurring in the optical dielectric loss (ε_i_) spectra [83,84,85,86,87,88]. Hence, as shown in Figure 12, the intercept of linear parts of optical dielectric loss spectra with the photon energy axis can yield the real energy gap. This is associated with the close correlation between the optical dielectric function and the electron-photon interaction and links the interband transition physical process within the solid electronic structure.

The imaginary component (ε_i_) of dielectric function primarily defines the electron transition from occupied to unoccupied states [81,87]. As validated by a previous work, by investigating of the imaginary part of the optical dielectric function, the optical transition mechanism could be grasped intensively [83]. A photon has the ability to excite an electron from an occupied state in the valence band to an unoccupied state in the conduction band, which is known as an interband transition. Of a quantum mechanical nature [88], this process involves photon absorption, formation of an excited electronic state and the leaving behind of a hole. From the perspective of quantum mechanics (microscopic), there is a close correlation between the optical dielectric loss and both the occupied and unoccupied electronic states in a solid. Furthermore, it has been microscopically (quantum mechanically) confirmed that correspondence exists between the primary peak in the imaginary part of the dielectric function and robust interband transitions [70,81,87].

**Figure 13:**
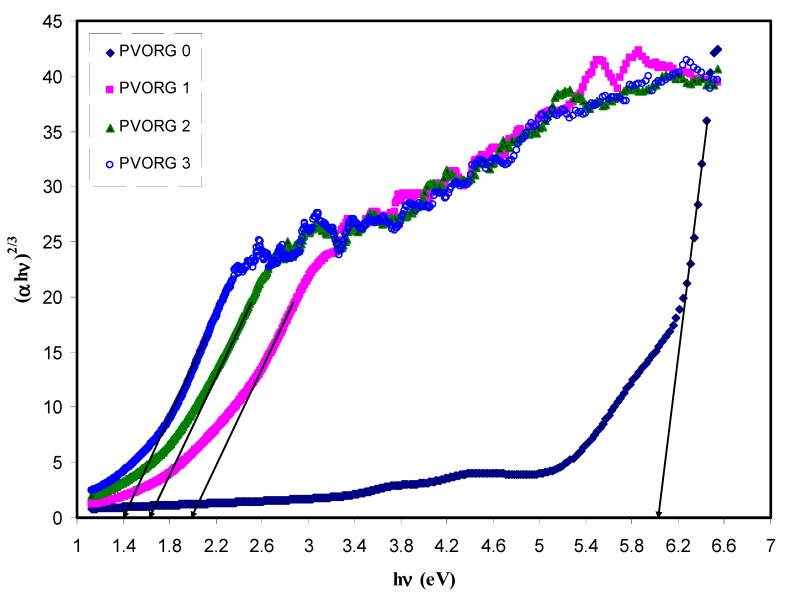
Plot of *(αhυ)^2/3^* vs. photon energy for pure PVA (PVORG0) and PVA composite films.

**Figure 14:**
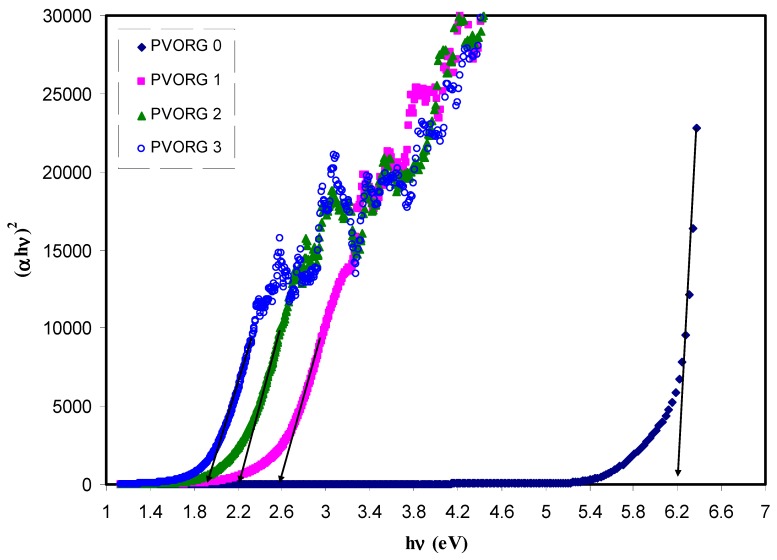
Plot of *(αhυ)*^2^ vs. photon energy for pure PVA (PVORG0) and PVA composite films.

In addition to facilitating analysis of optically-induced transition, the optical absorption method is also informative regarding the band structure of materials [61]. Fundamental absorption alludes to band to band transitions governed by specific selection rules and it manifests itself by a quick increase in the fundamental absorption region [89,90]. Depending on the material band structure, there can be a number of types of transitions [90]. The band gap (*E_g_*) represents the value of optical energy that an electron has to absorb in order to surpass the gap between the valence band and conduction band. The material band gap can be measured based on the fundamental absorption equivalent to the transition from valence band to conduction band. The equation for characterising the absorption in cases of photon energies (hv) exceeding the fundamental absorption edge is [91,92]:

*αhv* = *B*(*hv* − *E_g_*)^γ^ (10)

where *B* is a constant associated to the extent of the band tailing, and *hv* is the energy of incident photon.The value of the power coefficient denoted by parameter γ is calculated according to the types of possible electronic transitions, namely, 1/2 for direct allowed, 3/2 for direct forbidden, 2 for indirect allowed or 1/3 for indirect forbidden [77,81,93]. The plot of (αhν)^1/γ^ against photon energy (hυ) for the exponents (γ = 3/2 and 1/2) values of Tauc’s equation is illustrated in Figure 13 and Figure 14. Furthermore, the plotting of (αhν)^1/γ^ against hν can help to establish possible transitions by extrapolating the straight line portion of the graph on the hν axis to *α* = 0 and thus obtaining the equivalent band gap. The optical band gap values from Tauc’s method and optical dielectric loss plot are listed in Table 2.

It is clear from Table 2 that the estimated band gap values for composite samples for γ = 3/2 (direct forbidden) is close enough to those obtained from the optical dielectric loss plot (Figure 12), meaning that the crystalline structure is perturbed in hybrid samples. Therefore, the direct forbidden (γ= 3/2) results for hybrid samples is associated to the reduce of crystalline order in PVA composite films. The band gap achievedfrom Figure 13 and Figure 14 for different values of γ from Tauc’s equation [Equation (10)] in comparison to *E_g_* value obtained from optical dielectric loss plot indicates that the nature of electronic transition in pure PVA is direct allowed (γ =1/2).

From the perspective of solid-state physics, an energy band gap represents an energy range in a solid where the existence of any energy levels is impossible. This implies the lack of free carrier absorption and that the importance of interband transitions is restricted to cases of relatively high photon energies [80]. In the current study, the band gap derived for the samples is relatively small and similar to those obtained for inorganic semiconductors and organometallic-based polymers. A value of 2.5 eV has been attributed to the band gap for conductive polymers like Poly[2-methoxy-5-(2-ethyl)hexoxy-1,4-phenylenevinylene] (MEH-PPV), which is among the conducting polymers with the greatest potential for different optoelectronic applications (e.g., organic light-emitting diodes, sensors and organic solar cells) because it is environmentally stable, its conductivity can be easily controlled, it is not difficult to process, and can be produced in large amounts cost-effectively [64]. Therefore, according to the outcomes of the band gap analysis, the green remediation method is suitable for fabricating polymer hybrids that have a small optical band gap.

## 3. Materials and Methods

The black tea leaves were procured from a nearby market and distilled water was used to extract the natural colorant tea solution from the black tea leaves. This extraction process involved the addition of 50 g of black tea leaves to 250 mL distilled water at a temperature of 90 °C whilst avoiding exposure to direct sunlight. After 10 min the extract solution of black tea was separated. For this purpose, Whatman filter paper (Whatman 41, cat. No. 1441) with 20-µm pore size was used to eliminate the residues through filtration. At the same time 10 g of copper chloride (CuCl_2_) [CAS Number 7447-39-4, Molecular Weight = 134.45, Merck] was dissolved in 200 mL of distilled water. The preparation of organocopper-based material was carried out by addition of dissolved CuCl_2_ to the natural colorant tea solution which is at 80 °C. The solution was stirred for 10 min. The organocopper-based material was confirmed to have formed when the dark-coloured tea solution became green and precipitation manifested as clouds at the bottom of the beaker. The solution containing Cu metal complex was left to cool down to room temperature. Distilled water was used to wash the Cu metal complexes several times. Subsequently, the Cu metal complexes were dispersed in 100 mL distilled water.

Sigma Aldrich supplied (Sigma-Aldrich, Kuala Lumpur, Malaysia) the employed Poly (Vinyl Alcohol) (PVA) powder material. The well-known solution cast technique was used for the preparation of the solid polymer (SP) films based on PVA incorporated with Cu metal complex. Preparation of the PVA polymer solution involved addition of distilled water to PVA powder, followed by 60-min stirring with a magnetic stirrer at 80 °C. The PVA solution was then allowed to cool down to ambient temperature. This was followed by separate addition of 15 to 45 mL Cu metal complex solution to the homogeneous PVA solution in 15–mL steps. The mixtures were constantly stirred for 50 min. The samples were coded as PVORG0, PVORG1, PVORG2 and PVORG3 for PVA filled with 0, 15, 30 and 45 mL of Cu metal complex solution. For the purposes of film formation, the solutions were cast on dry Petri dishes and allowed to dry at an ambient temperature. Prior to characterisation, the films were placed in a desiccator with blue silica gel for further drying.

### 3.1. X-ray Diffraction

An X-ray diffractometer (Bruker AXS, Billerica, MA, USA) of 40-kV operating voltage and 45-mA current was employed to measure the X-ray diffraction (XRD) at ambient temperature. A beam of monochromatic, X-radiation of wavelength *λ* = 1.5406 A° enabled scanning of the samples, with the glancing angles being in the range 5° ≤ 2*θ* ≤ 90° and 0.05° step size.

### 3.2. Fourier Transform Infrared (FTIR) Spectroscopy

The pure PVA and doped PVA samples were analysed based on an FTIR spectrophotometer (Thermo Scientific, Nicolet iS10, (Perkin Elmer, Waltham, MA, USA) in the wavenumber region 4000–400 cm^−1^ with 2 cm^−1^ resolution.

### 3.3. UV- vis Measurement

A Jasco V-570 UV-Vis-NIR spectrophotometer (Jasco SLM-468, Tokyo, Japan) in absorbance mode was employed to record the ultraviolet-visible (UV-vis) absorption spectra of the solid polymer films based on PVA.

## 4. Conclusions

In this work, a new approach was used to transfer hazardous and dangerous heavy metals to inorganic or organometallic-based complexes using green chemistry remediation. This work shows an innovative approach for fabricating flexible polymer hybrids with small optical band gaps close to inorganic-based semiconductors. The novel ideas about transition metal salts and green chemistry approaches have been introduced and the findings obtained can lay the foundation for a new research domain by advancing knowledge regarding polymer science and organometallic complexes. Involving the use of black tea extract solution, the proposed approach of heavy metal remediation demonstrates sustainability and therefore has potential for global environmental sciences and engineering. The structural study indicates that black tea contains sufficient functional groups and conjugated double bonds. The UV-vis and FTIR study validated organocopper synthesis. Moreover, XRD and FTIR analysis indicate that complexation occurred between organocopper and PVA host matrix. The increase of the amorphous phase is reflected in the wideness increase and lessening in the XRD pattern intensity. The band shifts and reduces intensity in the PVA doped polymers are illustrated through the FTIR technique. The optical parameter investigation reveals that PVA-based hybrids have a small optical band gap close to inorganic-based materials. The shifting of absorption edge to lower photon energy was observed. The refractive index greatly tuned upon addition of organocopper to PVA polymer. Taucs model was used to study the type of electronic transition in hybrid materials. To estimate the optical band gap, the optical dielectric loss was studied precisely. Owing to their optical band gap and flexibility, these samples have great potential for broad use in optoelectronic devices. Additionally, it is suggested that the limitations presented by conjugated polymers could be addressed through the use of polymer hybrids demonstrating satisfactory ability for film formation. A comprehensive discussion of the relationships between structure and properties was extended on the basis of the XRD outcomes and band gap values.

## Figures and Tables

**Table 1:** Absorption edge values for pure PVA (PVORG0) and PVA composite films.

Sample Code	*Absorption Edge* (eV)
PVORG0	6.21
PVORG1	2.04
PVORG2	1.62
PVORG3	1.41

**Table 2:** Opticalbandgap from Tauc’s method and optical dielectric loss plot.

Sample Code	*E_g_* for *γ* = 1/2	*E_g_* for *γ* = 3/2	*E_g_* from *ɛ_i_* Plot
PVORG0	6.2	6.06	6.2
PVORG1	2.6	2	2
PVORG2	2.2	1.61	1.6
PVORG3	1.9	1.4	1.4
